# Renal Dysfunction, Metabolic Syndrome and Cardiovascular Disease Mortality

**DOI:** 10.1155/2010/167162

**Published:** 2010-03-24

**Authors:** David Martins, Chizobam Ani, Deyu Pan, Omolola Ogunyemi, Keith Norris

**Affiliations:** Department of Medicine, Charles Drew University of Medicine and Science, 1731 E 20th Street, Los Angeles, CA 90059, USA

## Abstract

*Background*. Renal disease is commonly described as a complication of metabolic syndrome (MetS) but some recent studies suggest that Chronic Kidney disease (CKD) may actually antecede MetS. Few studies have explored the predictive utility of co-clustering CKD with MetS for cardiovascular disease (CVD) mortality. *Methods*. Data from a nationally representative sample of United States adults (NHANES) was utilized. A sample of 13115 non-pregnant individuals aged ≥35 years, with available follow-up mortality assessment was selected. Multivariable Cox Proportional hazard regression analysis techniques explored the relationship between co-clustered CKD, MetS and CVD mortality. Bayesian analysis techniques tested the predictive accuracy for CVD Mortality of two models using co-clustered MetS and CKD and MetS alone. *Results*. Co-clustering early and late CKD respectively resulted in statistically significant higher hazard for CVD mortality (HR = 1.80, CI = 1.45–2.23, and HR = 3.23, CI = 2.56–3.70) when compared with individuals with no MetS and no CKD. A model with early CKD and MetS has a higher predictive accuracy (72.0% versus 67.6%), area under the ROC (0.74 versus 0.66), and Cohen's kappa (0.38 versus 0.21) than that with MetS alone. *Conclusion*. The study findings suggest that the co-clustering of early CKD with MetS increases the accuracy of risk prediction for CVD mortality.

## 1. Introduction

Cardiovascular disease (CVD) remains the leading cause of death and disability in the United States, accounting for approximately 1 of every 2.8 deaths recorded in the United States [1]. The tendency for the major CVD risk factors to occur in clusters informed considerable research interest leading to the description of Metabolic Syndrome (MetS) [2]. The National Cholesterol Education Program (NCEP) Adult Treatment Panel (ATP) III accepts the cooccurrence of abdominal obesity, atherogenic dyslipidemia, elevated blood pressure, insulin resistance with or without glucose intolerance, prothrombotic states, and proinflammatory states as MetS [3]. Although the precise pathophysiological mechanism of MetS is unknown, there is ample evidence of the association of this syndrome with CVD and CKD. 

CKD is the ninth leading cause of death in the United States with an estimated 19 million U.S. adults (6%) reported to have some form of CKD [4]. The association of several components of MetS with CKD [5–8] has engendered the perception of CKD as a long-term complication of MetS. However, the establishment of hyperinsulinemia and glucose intolerance as the pathophysiological bases for insulin resistance in patients with renal disease along with the recent implication of the renin-angiotensin system in local pancreatic islet structure and function suggests a plausible common pathophysiological mechanism for MetS and CKD [9, 10]. 

CKD is an established independent predictor of CVD mortality and a long-term complication of several of the individual components of MetS. However, the value of the diagnosis of MetS as an independent predictor of CVD mortality is controversial [2, 11]. A recent review of existing prospective data concludes that the CVD risk associated with the diagnosis of MetS varies with the components employed in the diagnosis of the syndrome and that the diagnosis of MetS itself only modestly predicts (Relative Risk 1.65–1.93) CVD mortality [12, 13]. Many of the components of MetS are also established CVD risk factors and the clinical challenge has always been the establishment of the additional risk posed by the diagnosis of MetS above and beyond the sum of the CVD risk factors employed in its diagnosis.

We hypothesize that the inclusion of indices of CKD in the diagnostic criteria for MetS will refine its identity as a clinical entity and improve its predictive value for CVD mortality. The purpose of our study is to explore the effect of including indices of CKD as defined by the National Kidney Foundation Kidney Disease Outcomes Quality Initiative (KDQI) study [14] in the diagnostic criteria for MetS on the predictive value of the syndrome for CVD mortality in a nationally representative sample.

## 2. Subjects and Methods

This study utilized data from a nationally representative sample of the civilian, noninstitutionalized US population collected by the Centers for Disease Control and Prevention during the third National Health and Nutrition Examination Surveys (NHANES III) conducted at 89 survey locations between January 1, 1988, and December 31, 1994. This survey utilized a complex multistage cluster design and over-sampled persons 60 years and older, non-Hispanic black individuals, and Mexican American individuals to enhance the precision of prevalence estimates in these groups. In-person interviews were conducted in sampled households, and all subjects were invited to participate in medical examinations conducted at a nearby NHANES III mobile examination center. Interviews consisted of demographic, socioeconomic, dietary, as well as health-related questions, and the mobile examination component consisted of medical and dental examinations, physiological measurements, and laboratory tests. The prevalence of common chronic conditions and associated risk factors was also determined during this survey. Details of the survey design and examination procedures have been previously published [15, 16]. The primary study outcome, CVD mortality was recorded from the NHANES III mortality follow-up data. This mortality follow-up data relied on a probabilistic match between NHANES III and National Death Index (National Center for Health Statistics (NCHS) 2006) death certificate records available for a total of 18,149 participants of the total 30,818 (59%) adult participants (those 17 years and older) that completed the initial interviews and physical examination and laboratory assessment at a mobile examination center [17]. Mortality assessments were conducted from the baseline interview in 1988–1994 through the end of the follow-up period in December 31, 2000. These mortality data included cause specific mortality and mortality dates. Cause specific mortality was coded using the International Classification of Diseases Ninth Revision (ICD-9) Clinical Modification for deaths occurring between 1988 and 1998 and the International Classification of Diseases Tenth Revision for deaths occurring between 1999 and 2000. We selected the total sample of individuals with mortality assessment during the follow-up period (*n* = 18,149). Of these we excluded participants who were pregnant and less than 35 years of age to get a total sample size of 13115 for analysis.

## 3. Study Variables

### 3.1. Outcome Variable

CVD mortality was the primary outcome variable of interest. Using the NHANESIII ICD-9 codes for deaths occurring between 1988 and 1998 and the ICD-10 Tenth Revision for deaths occurring between 1999 and 2000 we created a dichotomous variable with categories for CVD mortality and no-CVD mortality [17]. 

### 3.2. Primary Predictor Variable

The primary predictor variable was cocustered MetS and CKD. To compute this variable we first defined the individual metabolic risk factors or conditions consistent with the National Cholesterol Education Program (NCEP) Adult Treatment Panel III (ATP III) classification among the sample [18]: specifically (a) Elevated waist circumference: Men ≥ 40 inches (102 cm) and Women, ≥ 35 inches (88 cm), (b) Elevated triglycerides: ≥150 mg/dL, (c) Reduced HDL cholesterol: Men ≤ 40 mg/dL or Women ≤ 50 mg/dL, (d) Elevated blood pressure: ≥130/85 mm Hg, (e) Elevated fasting glucose: ≥110 mg/dL, (f) Prothrombotic state: fibrinogen > 350 mg/dL, and (g) Proinflammatory state: C-reactive proteins: > 0.5 mg/dL. Then we identified study participants meeting a minimum of three of these criteria and classified them as having MetS. We also created a trichotomous CKD variable using the KDQI study classification for stages of CKD [19]. Individuals in stages 3–5 (eGFR of 0–59 mL/min/1.73 m^2^) were classified as having late CKD; individuals in the stages 1 and 2 (eGFR of 60–89 mL/min/1.73 m^2^ or eGFR of >90 mL/min/1.73 m^2^ with proteinuria) were classified as having early CKD. All other individuals were classified as having normal kidney function. Finally we created a dummy variable with six categories for all the possible permutations of renal dysfunction and MetS from the two variables described earlier. The categories were as follows: (a) No MetS and No CKD, (b) No MetS and Early CKD, (c) No MetS and Late CKD, (d) MetS and No CKD, (e) MetS and Early CKD, and (f) MetS and Late CKD.

### 3.3. Covariates

Covariates included factors demonstrated to be associated with CVD mortality and include socio-demographic, smoking status, history of CVD, and mortality follow-up duration. Specifically, demographic factors included gender, age was categorized as <65 years and ≥65 years of age, gender, race, ethnicity, and poverty/income ratio. Smoking status was categorized as never smoked, former smokers and current smokers. Individuals with a self-reported history of physician diagnosis of stroke, myocardial infarction, and congestive heart failure were categorized as having a history of CVD. The time from baseline assessment, mortality assessment or death was included in the analysis to control for any temporal influences of time on the outcome of interest (CVD mortality). 

## 4. Statistical Analysis

Descriptive analyses of all the variables utilized in the data analysis were conducted. We explored the distribution of the variables among categories of CVD and no CVD mortality. Using the Cox proportional hazard model in multiple survival analysis regression tests and controlling for the influence of time to mortality assessment from baseline we explored the Univariate relationship between the predictor variables and CVD mortality (including both censored and event categories). The time to event was considered the time from the baseline laboratory measures and survey measurement to the time of mortality assessment or death of participants. A final multivariable survival analysis model was then created using all the significant predictors of CVD mortality in the univariate models to examine the influence of MetS and CKD on CVD mortality. Tests were conducted to rule out collinearity prior to running the final multivariable regression analysis model including all the covariates noted above. All data analyses were conducted using SAS (version 8.0; SAS Institute Inc, Cary, NC) and SPSS (version 15.0). Statistical hypotheses were tested using *P* < .05 as the level of statistical significance. Finally we also utilized the Bayesian analysis technique using the Waikato Environment for Knowledge Analysis software (Weka 3.0) to test the predictive accuracy of a model including a cocustered MetS and early CKD variable when compared to a model including separate MetS and early CKD measures while adjusting for other predictors of CVD mortality identified in the Univariate analysis. 

## 5. Results

### 5.1. Sample Characteristics

The sociodemographic and clinical characteristics of the study sample are presented in Table 1. Among the sample about 53% were female and 40% were aged 65 years or older. About 22% met at least 3 criteria for MetS. About 9.5% of the sample had eGFR levels below 60 mL/min/1.73 m^2^ and 43.9% had eGFR between 60 and 89 mL/min/1.73 m^2^ or greater than 90 with some evidence of proteinuria. The mean mortality follow-up period was about 97 months and CVD specific mortality among the sample was about 11.8%. 

### 5.2. Distribution of CVD Mortality

The distribution of CVD specific mortality in the sample is presented in Table 2. Higher mortality rates were observed in individuals who were male (12.9%), aged greater than 65 years (25.3%), lived below the 100% federal poverty line (15.1%), had a history of CVD (33.0%), had eGFR below 60 mL/min/1.73 m^2^ (33.7%) or had late stage renal disease without cooccurring MetS (38.1%) closely followed by individuals with both late stage renal disease with cooccurring MetS (31.0%). 

### 5.3. Unadjusted Relationship: Cocustered CKD and MetS versus CVD Mortality

Univariate analysis using Cox proportional hazard analysis demonstrated that individuals aged ≥65 years or older, male, who had a history of CVD, were white and lived below the 100% federal poverty income line were more likely to had significantly greater hazard of mortality (Table 3). Using the cocustered CKD and MetS variables categories, the results demonstrated that in increasing order, early CKD without MetS (HR = 3.02, CI = 2.45–3.72), MetS and early CKD (HR = 4.14, CI = 3.36–5.09), MetS and late CKD (HR = 12.81, CI = 10.27–16.00), and finally late CKD without MetS (HR = 15.39, CI = 12.16–19.48) all had a statistically significant higher mortality hazard when compared to no MetS or CKD. Individuals with MetS and no CKD did not demonstrate any statistically significant difference in hazard for CVD mortality when compared with individuals with no MetS or CKD.

### 5.4. Adjusted Relationship: Cocustered CKD and MetS versus CVD Mortality

**Figure 1:**
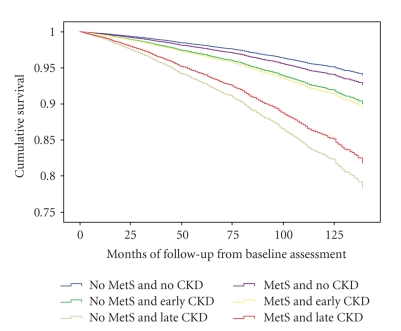
Survival function for CVD mortality versus cocustered metabolic syndrome and renal dysfunction. MetS stands for metabolic syndrome and CKD stands for chronic kidney disease.

**Figure 2:**
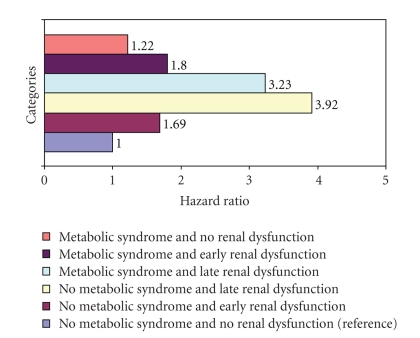
Bar chart: adjusted hazard ratios for cvd mortality versus categories of cocustered metabolic syndrome and renal dysfunction. OR for metabolic syndrome and no renal dysfunction not significant at *P* < .05.

After adjusting for other potential predictors of mortality a Cox proportional hazard model was developed to explore the relationship between cocustered CKD and MetS with CVD mortality. All measures reported previously in the Univariate analysis model as being significantly associated with CVD mortality remained the same (Table 3). Using the cocustered CKD and MetS variables categories, the results demonstrated that in the same increasing order as observed in the Univariate analysis, early CKD without MetS (HR = 1.69, CI = 1.37–2.10), MetS and early CKD (HR = 1.80, CI = 1.45–2.23), MetS and late CKD (HR = 3.23, CI = 2.56–3.70), and finally late CKD without MetS (HR = 3.92, CI = 3.06–5.03) all had a statistically significant higher mortality hazard when compared to no MetS or CKD. Individuals with MetS and no CKD did not demonstrate any statistically significant difference in hazard for CVD mortality when compared with individuals with no MetS or CKD. Figure 1 demonstrates the adjusted relative hazard for CVD mortality among the categories of cocustered CKD and MetS variable. Figure 2 presents a bar chart displaying the adjusted relative hazard ratios for CVD mortality among the categories of cocustered CKD and MetS variable. 

### 5.5. Predictive Accuracy of Models Cocustering Early CKD and MetS versus MetS Alone for CVD Mortality

The results of the Bayesian analysis as shown in Table 4 demonstrated that for CVD mortality a model with cocustering of early CKD and MetS demonstrated higher predictive accuracy (72.0% versus 67.6%), area under the ROC (0.74 versus 0.66), and Cohen's kappa (0.38 versus 0.21) than that with MetS alone.

## 6. Discussion

The poor predictive value of the diagnosis of MetS for CVD mortality has cast serious doubt on the existence of MetS as a clinical entity distinct from its diagnostic components. However, the clustering of metabolic abnormalities that led to the description of the syndrome is still apparent in clinical practice. This clustering of metabolic abnormalities more often than would be predicted by chance alone was the basis for the description of the syndrome and the suggestion of a common etiology for its components. The initial association of the syndrome with glucose intolerance and hyperinsulinemia led to the suggestion of Insulin resistance as the probable common pathophysiological basis for the syndrome [20–22]. However, subsequent conflicting series of studies led the National Cholesterol Education Program (NCEP) Adult Treatment Panel (ATP) III to conclude that the relationship between insulin resistance and MetS is not well established [3]. 

The emerging role of vitamin D as a modulator of both insulin resistance and the renin-angiotensin system [23] and the recent implication of the renin-angiotensin system in local pancreatic islet structure and function suggest a broader base for a probable common etiology for MetS than previously anticipated. The traditional association of CKD with several components of MetS may have led to the exclusion of CKD as a component of MetS and misled the search effort for a common pathophysiological basis for the components of the syndrome. It is in the light of this emerging body of evidence that we evaluate the effect of including early CKD in the diagnostic criteria for MetS on its predictive value for CVD mortality using a nationally representative sample. 

The preponderance of participants with early CKD in the analysis sample makes it suitable for the isolation of the influence of early CKD on the predictive value of MetS for CVD mortality. The association of CKD with CVD mortality in this study is consistent with the existing body of evidence and highlights the significance of CKD as an independent risk factor for CVD mortality. Even in the early stages CKD carries a significant risk for CVD mortality (OR 1.69; CI 1.37–2.10) (Table 3). In the absence of early CKD, MetS carries no significant risk for mortality in this study. However, early CKD becomes more hazardous with a hazard ratio of 1.8 versus 1.69 for CVD mortality in the presence of MetS (Figure 2). The greater hazard of early CKD in the presence of MetS is associated with a slight but similar reduction in survival among the affected participants (Figure 1). The apparent lack of a significant association between MetS and CVD mortality is consistent with the results of several previous studies but the increase in the hazard ratio for CVD mortality associated with early CKD in the presence of MetS gives credence to a potential renal contribution to the pathogenesis and perhaps mortality of MetS.

The primary purpose of this study was to explore the implication of including indices of early CKD in the diagnostic criteria of MetS for the predictive value of the syndrome for CVD mortality. The results of this study suggest a broader base for a common etiology for MetS and support the finding of a potential renal contribution to the etiology of the syndrome. The results of this study represent an analysis of a nationally representative sample of 13,115 participants with a mean mortality follow-up period of about 97 months and CVD specific mortality of 11.8%. 

However, the mortality follow-up data employed in this study relied on a probabilistic match between NHANES III and National Death Index (National Center for Health Statistics (NCHS) 2006) death certificate records available and inaccurate matches for CVD specific mortality may potentially confound the study findings, particularly since a few studies suggest that appropriately identifying CVD-specific mortality may inadvertently result in misclassification of cause specific mortality [24]. Also given the cross sectional nature of risk measures utilized in our study the impact of other confounding events post assessment may impact our finding and influence the attribution of CVD mortality to our study risk measures. 

Overall the study findings that early CKD may cocuster with MetS to result in an increased mortality burden should inform more studies to explore the temporal relationship between the pathogenesis of CKD and MetS.

## Figures and Tables

**Table 1:** Sample weighted distribution of study variables (*n* = 13115).

Variables	%
Race	
White	49.9%
African american	24.8%
Hispanic & other	25.4%
Gender	
Male	46.7%
Female	53.3%
Age (years)	
<65	60.0%
≥65	40.0%
Poverty/income ratio	
<1	36.3%
≥1	63.7%
Smoking status	
Never smoked	45.4%
Ever-smoker (currently nonsmoker)	31.4%
Current-smoker	23.1%
History of CVD (stroke, MI & congestive heart failure)	
No	86.9%
Yes	13.1%
Hypertriglyceridemia	
<150 mg/dL	59.3%
≥150 mg/dL	40.7%
Decreased HDL cholesterol (men ≤ 40 mg/dL/women ≤ 50 mg/dL)	
No	62.0%
Yes	38.0%
Blood pressure (≥130/85 mmHg)	
<110 mmHg systolic & 85 mmHg diastolic	36.8%
>130 mmHg systolic or >85 mmHg diastolic	63.2%
Diabetes (FBS, medications for DM & physician diagnosis)	
<110 mg/dL	74.9%
>110	25.1%
Central obesity (waist circumference: men ≥ 40 inches/women ≥ 35 inches)	
No	49.9%
Yes	50.4%
Proinflammatory state (CRP ≥ 3 mg/dL)	
No	73.0%
Yes	27.0%
Prothrombotic state (fibrinogen ≥ 350 mg/dL )	
No	71.7%
Yes	28.3%
Metabolic syndrome (meet at criteria for 3 or ATP III criteria)	
No	77.9%
Yes	22.1%
Renal dysfunction (eGFR in mL/min/1.73 m^2^ and proteinuria)	
0–59	9.5%
60–89 or eGFR ≥90 with proteinuria	43.9%
≥90 with no proteinuria	46.6%
Renal dysfunction & metabolic syndrome	
No metabolic syndrome and no renal dysfunction	31.9%
No metabolic syndrome and early renal dysfunction	24.4%
No metabolic syndrome and late renal dysfunction	3.6%
Metabolic syndrome and no renal dysfunction	14.7%
Metabolic syndrome and early renal dysfunction	19.4%
Metabolic syndrome and late renal dysfunction	6.0%
CVD specific mortality	
No	88.2%
Yes	11.8%
Interview/exam to mortality follow-up period in months (mean ± std)	96.5 [±33.0]

Sample excludes individuals aged <35 years, pregnant, or have no mortality assessment.

**Table 2:** Distribution of Independent Variables and Cardiovascular Disease Mortality (*n* = 13115).

Independent variables	No CVD mortality	CVD mortality
	frequency (%)	frequency (%)
Percentage of population	88.2%	11.8
Race		
White	84.5%	15.5%
African american	90.7%	9.3%
Hispanic & other	92.9%	7.1
Gender		
Male	87.1%	12.9%
Female	89.1%	10.9
Age (years)		
<64	97.2%	2.8%
≥65	74.7%	25.3
Poverty/income ratio		
<1	84.9%	15.1%
≥1	90.0%	10.0
Smoking status		
Never smoked	88.0%	12.0%
Ever-smoker (currently nonsmoker)	85.6%	14.4%
Current smoker	92.0%	8.0
History of CVD (stroke, MI & congestive heart failure)		
No	91.3%	8.7%
Yes	67.0%	33.0
Metabolic syndrome (meet at criteria for 3 or ATP III criteria)		
No	88.2%	11.8%
Yes	88.2%	11.8
Renal dysfunction (eGFR in mL/min/1.73 m^2^ and proteinuria)		
0–59	95.3%	33.7%
60–89 or eGFR ≥90 with proteinuria	89.8%	11.7%
≥90 with no proteinuria	61.9%	4.5
Renal dysfunction & metabolic syndrome		
No metabolic syndrome and no renal dysfunction	96.3%	3.7%
No metabolic syndrome and early renal dysfunction	89.8%	10.2%
No metabolic syndrome and late renal dysfunction	61.9%	38.1%
Metabolic syndrome and no renal dysfunction	93.9%	6.1%
Metabolic syndrome and early renal dysfunction	86.4%	13.6%
Metabolic syndrome and late renal dysfunction	69.0%	31.0%

**Table 3:** Univariate and Multivariable Cox Regression Analysis Cardiovascular Disease Mortality versus Independent Variables (*n* = 13115).

	Univariate analysis			Multivariable analysis		
	OR	95.0% C.I.	Sig.	OR	95.0% C.I.	Sig.
Race						
White (ref)	1.00	—		1.00	—	
African american	0.57	0.50–0.65	<.001	0.83	0.71–0.98	.02
Hispanic & other	0.42	0.36–0.48	<.001	0.69	0.59–0.82	<.001
Gender (ref)						
Female	1.00	—		1.00	—	
Male	1.21	1.09–1.33	<.001	1.40	1.23–1.59	<.001
Age (years)						
<64 (ref)	1.00	—		1.00	—	
≥65	3.42	3.20–3.70	<.001	2.62	2.40–2.85	<.001
Poverty/income ratio						
<1 (ref)	1.00	—		1.00	—	
>1	0.63	0.57–0.70	<.001	0.73	0.64–0.83	<.001
Smoking status						
Never smoked (ref)	1.00	—		1.00	—	
Ever-smoker (currently nonsmoker)	1.23	1.10–1.37	<.001	0.97	0.85–1.11	N/S
Current-smoker	0.64	0.55–0.74	<.001	1.07	0.89–1.28	N/S
History of CVD (stroke, MI & congestive heart failure)						
No (ref)	1.00	—		1.00	—	
Yes	5.10	4.60–5.66	<.001	2.40	2.11–2.72	<.001
Renal dysfunction & metabolic syndrome						
No metabolic syndrome and no renal dysfunction (ref)	1.00	—		1.00	—	
No metabolic syndrome and early renal dysfunction	3.02	2.45–3.72	<.001	1.69	1.37–2.10	<.001
No metabolic syndrome and late renal dysfunction	15.39	12.16–19.48	<.001	3.92	3.06–5.03	<.001
Metabolic syndrome and no renal dysfunction	1.71	1.31–2.22	—	1.22	0.94–1.60	—
Metabolic syndrome and early renal dysfunction	4.14	3.36–5.09	<.001	1.80	1.45–2.23	<.001
Metabolic syndrome and late renal dysfunction	12.81	10.27–16.00	<.001	3.23	2.56–3.70	<.001

**Table 4:** Area under the Curve (AUC) for CVD Mortality using unadjusted metabolic syndrome measure compared with cocustered metabolic syndrome and early stage renal disease.

	Area under the ROC	Accuracy	Cohen's Kappa	Mortality cases/total
Metabolic syndrome	0.66	67.6%	0.21	1551/13115
Metabolic syndrome and early renal dysfunction	0.74	72.0%	0.38	418/5604
